# Don’t seek, don’t find: The diagnostic challenge of Wernicke’s encephalopathy

**DOI:** 10.1177/0004563220939604

**Published:** 2020-07-13

**Authors:** Sara Kohnke, Claire L Meek

**Affiliations:** 1 Institute of Metabolic Science, Metabolic Research Laboratories, University of Cambridge, Addenbrooke’s Hospital, Cambridge, UK; 2 Department of Clinical Biochemistry, North West Anglia NHS Foundation Trust, Peterborough City Hospital, Peterborough, UK; 3 Wolfson Diabetes and Endocrinology Clinic, Cambridge University Hospitals, Addenbrooke’s Hospital, Cambridge, UK

**Keywords:** Wernicke’s encephalopathy, ophthalmoplegia, nystagmus, bariatric surgery, obesity, nutrition, deficiency

## Abstract

Wernicke’s encephalopathy is caused by thiamine deficiency and has a range of presenting features, including gait disturbance, altered cognitive state, nystagmus and other eye movement disorders. In the past, Wernicke’s encephalopathy was described almost exclusively in the alcohol-dependent population. However, in current times, Wernicke’s encephalopathy is also well recognized in many other patient groups, including patients following bariatric surgery, gastrointestinal surgery, cancer and pancreatitis. Early recognition of Wernicke’s encephalopathy is vital, as prompt treatment can restore cognitive or ocular function and can prevent permanent disability. Unfortunately, Wernicke’s encephalopathy is often undiagnosed – presumably because it is relatively uncommon and has a variable clinical presentation. Clinical biochemists have a unique role in advising clinicians about potential nutritional or metabolic causes of unexplained neurological symptoms and to prompt consideration of thiamine deficiency as a potential cause in high-risk patient groups. The aim of this review is to summarize the clinical features, diagnosis and treatment of Wernicke’s encephalopathy and to highlight some non-traditional causes, such as after bariatric surgery.

## Case report

A 26 year old woman presented to the department of surgery with persistent vomiting six weeks after private gastric sleeve surgery. During her admission, she was found to have nystagmus, imbalance and gait disturbance, which was sufficiently severe to interfere with activities of daily living, such as walking, reading and watching television. A presumptive diagnosis of Wernicke’s encephalopathy was made and a prolonged course of high dose IV vitamin B_1_ (Pabrinex) caused a gradual improvement in symptoms.

## Introduction

Wernicke’s encephalopathy (WE), an acute neurological disorder caused by thiamine (vitamin B_1_) deficiency, is under-recognized and under-treated. Around 80% of patients with the condition do not receive a diagnosis, and many cases are only diagnosed postmortem. The delay in identifying and treating the condition can be clinically disastrous as, untreated, WE can lead to permanent neurological damage, psychiatric sequelae and death.

WE can be challenging to diagnose clinically or biochemically. Presentation of one or more of the classic triad of symptoms (described in Box 1) is highly suggestive of the condition, but these features are not always present. Previous studies found nearly a fifth of confirmed WE cases did not display any of these symptoms.^1^

Box 1. Classical symptoms of Wernicke’s encephalopathy.

1. Altered mental state and/or memory deficit

2. Nystagmus, ophthalmoplegia or other disordered eye movements

3. Ataxia/gait disturbance

Although classically, WE was described in the alcohol-dependent population, there is increasing awareness that other groups of patients, with no history of alcohol dependence, can also suffer from the condition. WE should be considered in patients with other conditions affecting nutrition, including hyperemesis gravidarum and following bariatric surgery.

## Terminology

The disease entity known as WE refers specifically to the acute neurological disorder caused by thiamine deficiency and is associated with the ‘classic triad’ of symptoms (Box 1). Approximately half of patients who survive WE will go on to develop chronic neuropsychiatric symptoms caused by thiamine deficiency, which is known as Korsakoff’s syndrome.^2,3^ While the symptoms of WE can resolve with appropriate treatment, Korsakoff’s syndrome is largely irreversible and is characterized by global amnesia, confabulation, apathy and disordered cognition.^3^ Due to the common progression of WE to Korsakoff’s syndrome, WE is sometimes referred to as ‘Wernicke–Korsakoff syndrome’.

Thiamine deficiency can also cause other systemic disorders. ‘Wet’ beriberi affects the cardiovascular system and can cause congestive heart failure. ‘Dry’ beriberi refers to nervous system damage caused by thiamine deficiency and includes polyneuropathy and WE.^4,5^ Although dry and wet forms of beriberi can occur in the same patient at the same time, this seems to be rare.^6^

## Epidemiology

**Figure 1.**
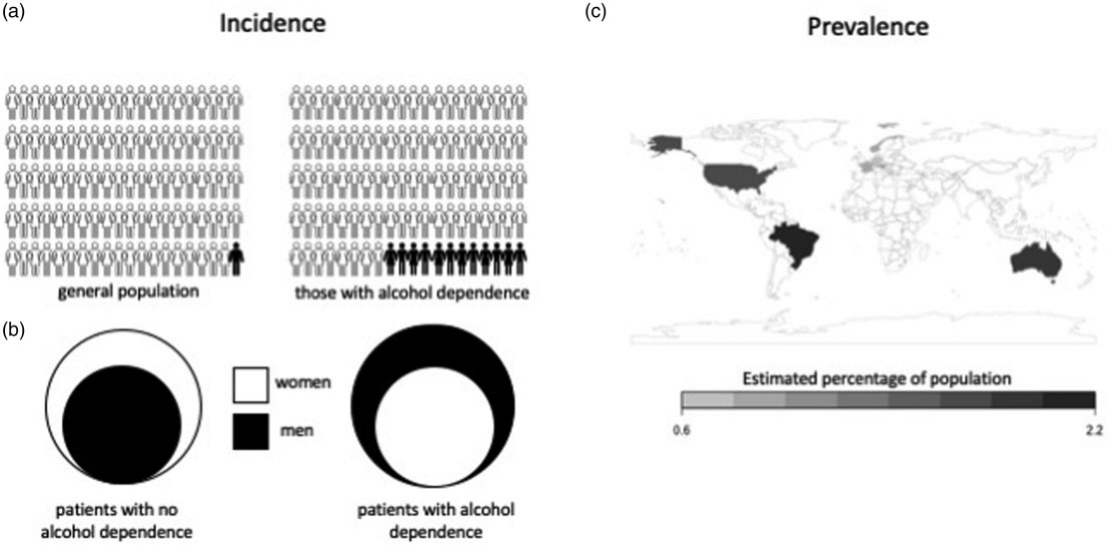
Epidemiology of WE. (a) Incidence of WE is estimated to be less than 1% in the general population and 12.5% in alcohol-dependent patients. (b) The ratio of males and females with WE differs depending on cause of the disease. For non-alcohol-related causes, females outnumber males 1.69:1, while males with alcohol-related WE outnumber females 1.84:1. (c) Australia, Austria, Brazil, France, Germany, Norway and the United States have documented the prevalence of WE based on autopsies with average ranges from 0.6 to 2.2%.

Postmortem histological analyses have provided evidence that WE occurs in about 1% of the general population and in 12.5–35.0% of alcohol-dependent patients.^7–9^ Data about WE prevalence throughout the world are limited, but available information indicates that the country-wide prevalence of the disease is not linked to per capita alcohol consumption (Figure 1).^5,10,11^ WE due to alcohol misuse is more common in males (1.69 males for each female patient), while non-alcohol related causes are more common in females (1.84 females for each male patient). The age of onset of WE also differs with disease causation. The average age of onset of the condition in alcohol-dependent patients is over 40 years old, while non-alcohol related causes are more common in younger age groups.^2,12^

## Thiamine deficiency and human disease

Unlike plants and microbes, animals are unable to synthesize thiamine and thus are reliant on dietary sources to meet requirements. Humans require between 1 and 2 mg of thiamine daily from the diet and have total body thiamine stores of around 30–50 mg. Therefore, stores can be exhausted anywhere between 18 days and 6 weeks with a thiamine-deficient or -devoid diet.^13,14^ Compared to other animals, humans have much lower brain thiamine concentrations: there is a 20 pmol/mg concentration of the bioactive form of thiamine in human brains, while certain primates have double this concentration and rodents have concentrations greater than 115 pmol/mg.^15,16^ The susceptibility of humans to thiamine deficiency is well-established, and many countries actively fortify foods such as bread and grains with thiamine.^17,18^

Although healthy people are susceptible to thiamine deficiency, certain medical conditions can increase the chances of developing thiamine deficiency and WE. These conditions are associated with decreased access, absorption, storage capability, impaired cellular utilization of thiamine, or increased metabolism or loss of thiamine.

## The pathology of thiamine deficiency

### Mechanisms of pathological sequelae from thiamine deficiency

**Figure 2.**
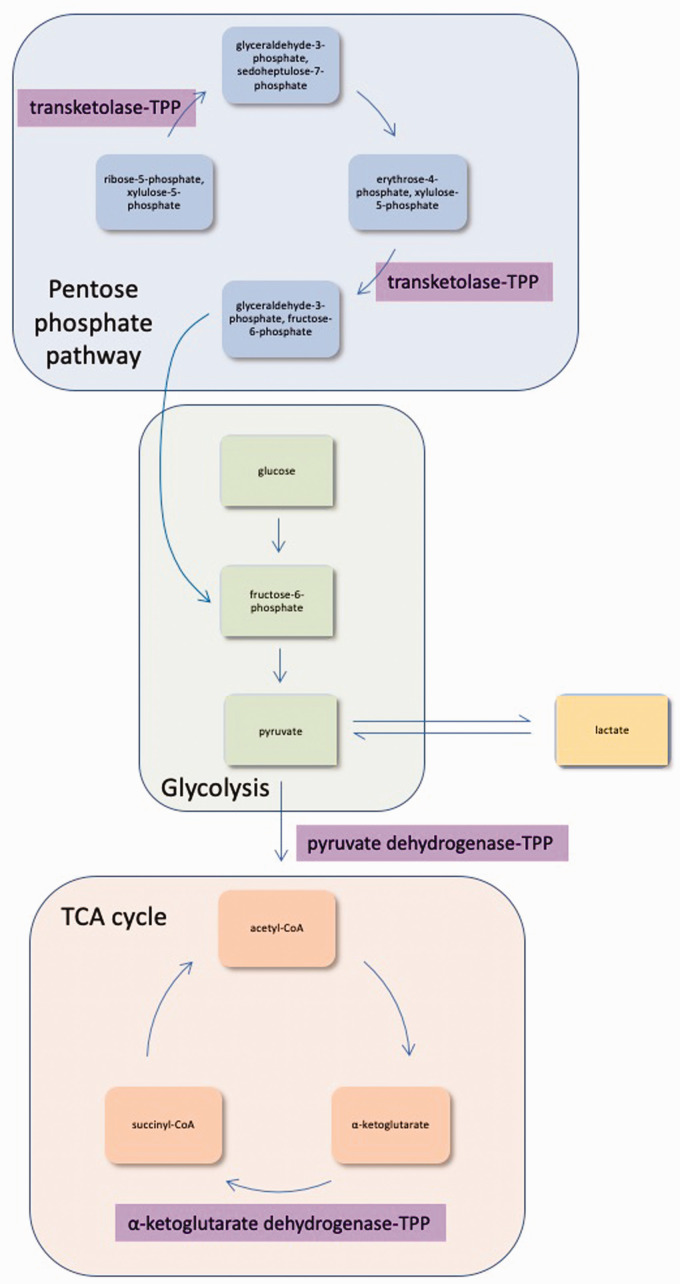
The bioactive form of thiamine is necessary for several cellular processes. TPP is the bioactive form of thiamine. It is a necessary cofactor in the pentose phosphate pathway, glycolysis and the TCA cycle. Additionally, it plays a role in maintaining equilibrium between pyruvate and lactate. TCA: tricarboxylic acid; TPP: thiamine pyrophosphate.

WE results from a lack of thiamine (vitamin B_1_) availability. Thiamine in its bioactive form thiamine pyrophosphate (TPP, also called thiamine diphosphate) is necessary for energy metabolism in all cells. TPP acts as a cofactor for transketolase in the pentose phosphate pathway, as a cofactor for pyruvate dehydrogenase in the transition from glycolysis to the tricarboxylic acid (TCA) cycle and as a cofactor for α-ketoglutarate dehydrogenase within the TCA cycle (Figure 2). Thiamine deficiency therefore disrupts cellular metabolism in several ways and limits the availability of ATP. It is thought the brain is the main site of damage due to its immense energy requirement compared to the rest of the body.^19,20^

The exact cause of brain damage in WE is unclear, but may be related to focal lactic acidosis, disruption of the blood–brain barrier, neural cell excitotoxicity, inflammation or insufficient amounts of cellular ATP.

Brain lesions in WE are often attributed to focal lactic acidosis.^5,21,22^ When thiamine is not available to facilitate pyruvate conversion through the TCA cycle, pyruvate accumulates within the cell. The concentrations of pyruvate and lactate are normally at equilibrium, and an increase in pyruvate causes a subsequent increase in lactate concentration (Figure 2).^23^ Increased lactate from thiamine deficiency causes a fall in pH in specific parts of the brain which are also affected in experimental animal models of WE.^24,25^

The idea that disruption of the blood–brain barrier may be a cause of WE lesions was first introduced in 1949.^26^ Increased permeability of the blood–brain barrier can allow large proteins not normally found in the central nervous system to pass into the brain and puts neurological tissue at risk of toxic effects. Several regions of the brain typically found to have lesions in WE are at blood–brain barrier junctions.^27^

Another proposed mechanism includes neural cell excitotoxicity, which is caused by extracellular build-up of glutamate. In thiamine deficiency, glutamate transporters in astrocytes (which normally clear the synapse of released glutamate by facilitating astrocyte uptake of the neurotransmitter) are downregulated, leading to sustained depolarization of neurons and subsequent death of the cells.^28,29^ Inflammation is also known to occur in thiamine deficiency, with microglial reactivity and pro-inflammatory cytokines found throughout the brain.^30^ However, the pathological effects of this are unknown.

## Medical conditions associated with WE

WE is classically considered a disease of alcohol-dependent patients. However, there are many other conditions that increase the likelihood of developing thiamine deficiency in patients with no history of alcohol misuse. These conditions are associated with decreased access, absorption, storage capability or cellular utilization of thiamine, or increased metabolism or loss of thiamine.

### Conditions of decreased access to thiamine

Thiamine deficiency and subsequent WE can occur when a person’s diet is deficient or devoid of thiamine. For example, WE has been documented after food deprivation during anorexia nervosa, religious fasting and malnutrition in the elderly.^31–33^ Lack of thiamine supplementation during total parenteral nutrition is another cause of WE.^34^ Conditions that cause nausea and vomiting are also linked to development of WE: hyperemesis gravidarum is a well-documented cause of the condition.^35,36^

### Conditions of decreased gastrointestinal (GI) absorption of thiamine

**Figure 3.**
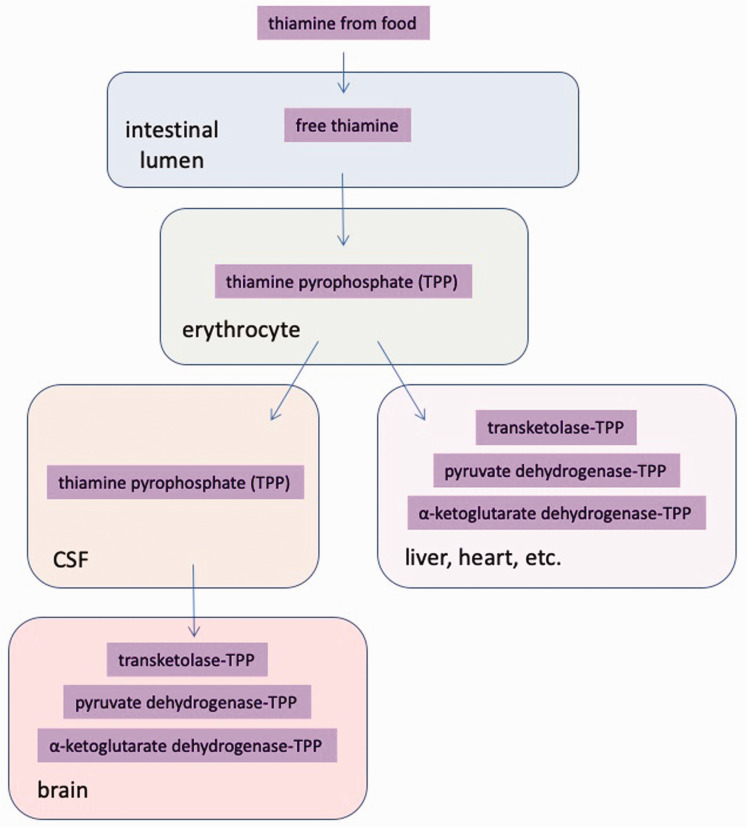
Modification and transport of thiamine from food. Thiamine derived from food sources is hydrolysed into free thiamine in the lumen of the intestine. Free thiamine is passed through the intestinal wall to erythrocytes where it is converted to the bioactive form of thiamine – TPP. Blood carries TPP to organs such as the liver, heart, etc. To pass into the brain, TPP must cross the blood–brain barrier into the cerebrospinal fluid of the central nervous system before it diffuses throughout the brain. CSF: cerebrospinal fluid.

Thiamine from food is hydrolysed into free thiamine by phosphatases in the lumen of the intestine.^17^ Free thiamine is then absorbed by the mucosa of the small intestine and converted to TPP in erythrocytes, then carried to stores in the liver, skeletal muscle, heart and kidney (Figure 3).^37–40^ In experimental animals, alcohol use has been shown to directly inhibit thiamine absorption in the GI tract.^41,42^ In humans, conditions that commonly disrupt nutrient absorption include Crohn’s disease, pyloric stenosis, peptic ulcers or chronic diarrhoea, which have all been linked to development of WE.^43–46^

Bariatric and GI surgery accounts for a large proportion of cases of WE due to decreased thiamine absorption.^2^ Cases of WE after bariatric surgery for weight loss have greatly increased in the past two decades.^47^ These operations often lead to both decreased access to thiamine and decreased absorption of thiamine. Patients often experience postoperative vomiting which reduces thiamine intake, and certain procedures, like Roux-en-Y gastric bypass, reduce thiamine absorption by reducing the length of the gut available for nutrient absorption.^48^

### Conditions of decreased thiamine storage capability

Thiamine is mainly stored in the liver.^49,50^ Conditions such as end-stage chronic liver failure are associated with depleted stores of thiamine and can lead to WE.^51^ This phenomenon may be exacerbated by regular alcohol use, as long-term administration of alcohol to experimental animals has been shown to cause diminished thiamine stores in both the brain and liver.^52^

### Conditions of impaired cellular thiamine utilization

Genetic variants may cause disruption to cellular utilization of thiamine. The majority of studies on genetic susceptibility to WE have focused on heritable dysfunctions in the transketolase enzyme. This enzyme binds with TPP in the pentose phosphate pathway (Figure 2). In some patients with WE, transketolase activity is impaired in likely a heritable manner, possibly by altered affinity of the enzyme to TPP.^53,54^ In those with altered transketolase binding, even vitamin supplementation may not be enough to overcome reduced transketolase activity.^54,55^

### Conditions of increased use/disposal of thiamine

Many patients with WE will have more than one contributing cause of thiamine deficiency, such as reduced thiamine intake and increased thiamine requirements. However, certain conditions cause increased metabolism or loss of thiamine itself. A large dietary carbohydrate load, a hypermetabolic state (such as hyperthyroidism) or certain rapidly growing cancers increase thiamine requirements and cause rapid depletion of body thiamine stores.^56–58^ Additionally, haemodialysis or peritoneal dialysis can promote accelerated loss of water-soluble vitamins such as thiamine.^59,60^ Administration of B vitamins should be considered in high-risk patients, including those on dialysis and when refeeding a malnourished patient.

## WE – a challenging diagnosis

WE is challenging to diagnose and many cases are identified on postmortem examination.^5,56^ Presentations of the disease vary widely between cases, and often the signs are subtle or absent.

### Clinical signs

The diagnosis of WE can be made clinically, based on the presence of the ‘classical triad’ of symptoms (Box 1). Caine and colleagues developed a proposed set of criteria for WE diagnosis which requires at least two of the following features to be eligible for diagnosis:

- Dietary deficiency
- Eye signs
- Cerebellar signs
- Mild memory impairment or altered mental state.

Caine et al.^61^ reported a sensitivity of 94% and specificity of 99% for the diagnosis of WE when these criteria were used. However, clinical presentation can differ greatly in individuals with WE. One study showed only 44% of those with postmortem WE diagnosis displayed two or more operational criteria before death.^1^ Due to the common absence of clinical signs, several other methods are employed to aid diagnosis.

### Biochemistry

Testing whole blood or erythrocytes for thiamine content is a very useful confirmatory test for thiamine deficiency in patients with suspected WE. However, these tests are not available in most clinical laboratories and treatment cannot be delayed to wait for the result. Ideally, when the diagnosis is suspected, blood draw before treatment is given is likely to maximize the likelihood of obtaining a diagnostically useful result.^10^

TPP, the active form of thiamine, is carried from the intestine to thiamine-storing organs via erythrocytes (Figure 3). Historically, assays for blood transketolase activity as an indirect measurement of TPP content have been used to assess thiamine deficiency.^62^ However, direct measurement of TPP or thiamine via high performance liquid chromatography has been shown to be more precise and robust.^63,64^ There is some controversy about reference ranges for thiamine, as some variation between regions is to be expected based on different dietary and environmental factors. Local laboratories normally obtain reference values from non-thiamine-deficient patients’ samples.^63,65^

### MRI scans

When WE is suspected in the absence (or even presence) of clinical signs, an MRI scan can be useful to assess if there are neurological abnormalities. As with clinical signs, the brain areas presented with damage in these scans vary widely from person to person. ‘Typical’ lesions found in MRI scans are seen in only 58% of patients. In these patients, an increased T2 signal (signifying oedema) can be found in the paraventricular regions of the thalamus and hypothalamus and the periaqueductal region, while the cerebellum and mamillary bodies may be reduced in size. To detect WE, MRI has a sensitivity of 53% but a specificity of 93%.^66^ Ideally, MRI will be performed before thiamine administration, as brain abnormalities are quickly reversed after treatment has begun.^67^ However, the need for urgent treatment often makes this impossible.

### Diagnosis at postmortem examination

As clinical signs are variable, the diagnosis of WE is often made postmortem. On macroscopic examination of the brain, shrunken and discoloured mamillary bodies are the most common abnormality, seen in around 80% of patients affected by WE. Atrophy of the cerebellum and dilation of ventricles are also common, and lesions are usually found near the ventricular system. Microscopically, common features of WE include increased numbers of blood vessels and gliosis in the mamillary bodies. Other histological features of brain tissue in affected patients include microhaemorrhages, gliosis, axon and myelin damage, and significant loss of cerebellar Purkinje cells.^68,69^

### Diagnostic challenges

It is often necessary to combine tests to increase the likelihood of making the diagnosis, but even multiple tests may not be enough to confirm a positive diagnosis. Treatment for WE is often begun before a diagnosis is confirmed. Many consider reversal of clinical signs upon treatment with thiamine to be the best support for an antemortem diagnosis of WE.^5^

## WE – a simple treatment

### Thiamine treatment

Due to the rapid progression of WE, it is recommended that therapeutic administration of thiamine be commenced in any case where thiamine deficiency is suspected, even before a firm diagnosis has been made. As patients may have impaired mechanisms to absorb the vitamin via the GI tract, parenteral administration is necessary.^13^ Vitamin B_1_ has an extremely low risk of adverse effects (anaphylactic shock was reported in four cases in one million intravenous administrations and one case in five million intramuscular administrations), therefore the potential gains from administration to a patient with possible thiamine deficiency far outweigh the risks of not treating the condition.^70^ There is no consensus on the dosing or duration of vitamin B_1_ to treat WE, although those with WE caused by alcohol use may need higher daily doses. The European Federation of Neurological Societies recommends intravenous administration of 200 mg thiamine three times daily until there are no additional improvements in clinical conditions, while British authors have recommended 500 mg three times per day for 2–3 days then 250 mg daily until improvements cease.^5,10^

### Vitamin B_1_ as prophylaxis

The use of vitamin B_1_ as prophylaxis is widespread internationally. Many countries fortify food with thiamine.^71^ Many hospitals use thiamine administration prophylactically for high-risk groups, including patients with malnutrition, hypoglycaemia or alcohol dependence.^10,60,72^ Recent studies have questioned this practice, however, providing evidence that there was no difference between administering thiamine before or after short-term glucose treatment.^72,73^ Patients with a history of bariatric surgery are advised to take prophylaxis indefinitely due to decreased ability to absorb thiamine. One author recommends doses of 50–100 mg orally three times per day.^74^

## Case report outcome

Our patient initially was treated with 250 mg thiamine intravenously three times daily (given as Pabrinex) which stabilized her symptoms but did not entirely resolve them. She responded more quickly to 500 mg thiamine three times daily, and her symptoms improved markedly over several weeks. Biochemistry and MRI results were inconclusive, but both tests had been initiated after the onset of treatment. The improvement to early administration of thiamine was considered consistent with the diagnosis, in a patient with recognized risk factors.

Early treatment did improve her symptoms, but they did not resolve entirely. At their peak, her symptoms were extremely disabling and included difficulties reading mobile phone messages or watching television. She had poor balance and could not walk unattended around the ward. Chronic disability appeared a likely long-term outcome. However, after many weeks of high dose thiamine treatment, her symptoms gradually subsided and she was able to start physiotherapy to improve mobility in preparation for discharge. Overall, her recovery was slower than expected, perhaps due to her frequent vomiting episodes which limited dietary thiamine replacement. Her vomiting episodes gradually settled with educational input about eating styles and food choices after bariatric surgery. She has now returned to work and remains on lifelong oral thiamine supplementation with regular nutritional follow-up in a specialist postbariatric clinic.

In general, our patient was very fortunate to avoid chronic neurological sequelae and long-term disability. Sadly, the prognosis in WE is often poor, with around 50% of patients having long-term consequences, including memory impairment or Korsakoff’s syndrome.^2,3^ In patients with WE due to alcohol dependence, around 80% will develop Korsakoff’s syndrome, and around 40% could die as a complication of Wernicke–Korsakoff syndrome.^2,12^ However, even among patients with a non-alcohol-related cause, only around 20% of patients make a complete recovery while a further 20% may die due to complications of the disease.

## Conclusions

WE is a serious and underdiagnosed disease. Historically thought to be a disease of alcohol misuse, it is now well recognized that many conditions can cause WE. WE after bariatric surgery is increasingly common and clinical biochemists have a unique opportunity to make sure the diagnosis is considered when a patient presents with unexplained neurological features. The diagnosis of WE is difficult and requires integration of clinical signs, MRI appearance and biochemistry results, and is complicated by the urgent need to start treatment before confirming the diagnosis. Thiamine is a safe and effective treatment and should be given prophylactically to high-risk groups. The prognosis for those who have experienced WE remains poor, especially for those with an alcohol-related cause, and relatively few patients make a full recovery.
